# Anti-Inflammatory Effect of *Artemisia argyi* on Ethanol-Induced Gastric Ulcer: Analytical, In Vitro and In Vivo Studies for the Identification of Action Mechanism and Active Compounds

**DOI:** 10.3390/plants10020332

**Published:** 2021-02-09

**Authors:** Myoung-Sook Shin, Jaemin Lee, Jin Woo Lee, Se Hoon Park, Il Kyun Lee, Jung A. Choi, Jung Suk Lee, Ki Sung Kang

**Affiliations:** 1 Korean Medicine, Gachon University, Seongnam-si, Gyeonggi-do 13120, Korea; ms.shin@gachon.ac.kr (M.-S.S.); jaemin.lee426@gmail.com (J.L.); 2 Research & Development Center, Richwood Pharmaceuticals, 1, Gwanak-gu, Seoul 08826, Korea; jinwoo0921@richwood.net (J.W.L.); parksehoon@richwood.net (S.H.P.); 1981ilkyun@richwood.net (I.K.L.); jjunga@richwood.net (J.A.C.); choiky@richwood.net (J.S.L.)

**Keywords:** *Artemisia argyi*, anti-inflammation, antigastric ulcer

## Abstract

*Artemisia argyi* is widely used as traditional medicine in East Asia. However, its effects against inflammation and gastric ulcers have not been reported yet. We analyzed anti-inflammatory activity and its molecular mechanisms of *A. argyi* using RAW264.7 cells line, then evaluated the curative efficacy in rats with acute gastric ulcers. Nitric oxide and IL-6 production was measured using Griess reagent and an ELISA kit. Inducible nitric oxide synthase (iNOS), interleukin (IL)-6, and mucin (MUC)1, MUC5AC, and MUC6 mRNA were determined by SYBR Green or Taqman qRT-PCR methods. The phosphorylation of ERK, JNK, p38, and c-Jun protein were detected by western blotting. RW0117 inhibited LPS-induced NO and IL-6 production. The mRNA levels of iNOS and IL-6 were strongly suppressed. The phosphorylation of ERK, JNK, and c-Jun decreased by treatment with RW0117. Oral administration of RW0117 recovered the amount of mucin mRNA and protein level that was decreased due to gastric ulcers by HCl-EtOH. *A. argyi* exhibited strong anti-inflammatory effects and contributed to the modulation of HCl-EtOH-induced gastric ulcer in rats.

## 1. Introduction

Inflammation is an immune response of a living organism against external stimuli or tissue damage, and it is a complex process involving various immune cells. However, excessive inflammatory reactions can cause chronic inflammatory diseases; corresponding responses to inflammatory reactions in the human body are closely related to the prevention or occurrence of diseases [1,2]. Phagocytosis or secretion of inflammatory mediators is a well-known inflammatory reaction by immune cells such as macrophages exposed to viruses or pathogens [3]. Macrophages play an important role in innate and acquired immune responses. Macrophages are activated by lipopolysaccharide (LPS) that produces inflammatory mediators such as nitric oxide (NO), prostaglandin E2, tumor necrosis factor-α (TNF-α), interleukin-6 (IL-6), and IL-1β [4,5]. LPS, also known as endotoxin, exists in the outer membrane of gram-negative bacteria and activates the transcription factor nuclear factor-κB (NF-κB), thereby activating inducible nitric oxide synthase (iNOS) and cyclooxygenase-2 (COX-2) and ultimately producing the respective inflammatory mediators [6]. Acute gastritis is a disease characterized by inflammation of the gastric mucosa due to stress, smoking, alcohol, irregular meals, or prolonged use of nonsteroidal anti-inflammatory drugs (NSAIDS) [7,8,9]. When this disease occurs, it causes gastrointestinal discomfort and loss of the gastric mucosa due to vomiting. The gastric mucosa has components such as mucus, bicarbonates, and prostaglandins that protect the stomach against gastric lesions [10]. In clinical practice, proton pump inhibitors and H2 receptor antagonists are used to treat ulcers, but these drugs cause side effects and recurrence of gastritis has been observed after treatment [11]. Alcohol is one of the most common pathogenic factors of gastric injury [7]. Excessive alcohol consumption can weaken the protective function of the gastric mucosa. Therefore, ethanol-induced gastric ulcer animal models are often used to screen for compounds that possess antiulcer activity [12,13,14]. Previous studies already reported that plant extracts and plant-derived compounds such as *Allophylus serratus,* Aloe vera and kaempferol (3,5,7,4′-tetrahydroxy flavone) showed anti-inflammatory and antigastric ulcer effects in in vitro and in vivo [15,16,17].

*Artemisia argyi (A. argyi)* has been traditionally used in China, Japan, and Korea to treat conditions such as inflammation, abdominal pain, hemorrhage, hepatitis, and liver cirrhosis [18]. Recent studies have shown that *A. argyi* extracts reduce symptoms of allergic asthma and dermatitis in vivo and possess immunosuppressive activity [19,20,21]. The flavonoids eupatilin, jaceosidin, hispidulin, and caffeoylquinic acid present in *A. argyi* are responsible for its pharmacological effects [22,23,24].

In this study, we prepared an extract of *A. argyi* (RW0117) and analyzed its anti-inflammatory activity and identified its intracellular signaling pathways. We also evaluated its antigastric ulcer activity in rats.

## 2. Materials and Methods

### 2.1. Chemicals and Reagents

Antibodies against p65 (C-20), p38 (C-20), ERK1 (C-16), JNK (FL), and β-actin (I-19) were purchased from Santa Cruz Biotechnology (Santa Cruz, CA, USA). Antibodies against phospho-p65 (Ser-536), phospho-ERK1/2 (Thr202/Tyr204), phospho-JNK (Thr-183/Tyr-185), phospho-p38 (Thr-180/Tyr-182), phospho-c-Jun (Ser-73), c-Jun (60A8), and inducible NO synthase (iNOS, D6B6S) were purchased from Cell Signaling Technology, Inc. (Danvers, MA, USA). Ultrapure LPS from *Escherichia coli* 0111:B4 was obtained from Invitrogen (San Diego, CA, USA). Dulbecco’s modified eagle’s medium (DMEM) was purchased from Hyclone (GE Healthcare Life Sciences, Chicago, IL, USA). Fetal bovine serum (FBS) was purchased from ATCC (Manassas, VA, USA). Eupatilin (CAS No.: 22368-21-4), jaceosidin (CAS No.:18085-97-7) and hispidulin (Cas No.:1447-88-7) were purchased from Sigma (St. Louis, MO, USA). All other chemicals and reagents were purchased from Sigma (St. Louis, MO, USA). Beeswax alcohol (Abexol^®^) was used as the positive control material and was purchased from Rainbow & Nature Pty Ltd. (Seoul, Korea).

### 2.2. Plant Material and Extraction

*Artemisia argyi H.Lev. & Vaniot (A. argyi)* was collected in Jiangsu Province in China (2018, June), and was purchased as dried material from NINGBO SIMINGSHAN BIO-TECHNOLOGY (Ningbo, Zhejiang, China) (2018, Sept.) Raw material was identified by comparing database of the National Center for Biotechnology Information (NCBI, USA) after DNA sequence analysis (Table S1). A total of 16 L of 66.5% ethanol was added to 1 kg of A. argyi leaves to perform cold extraction for 24 h. The extract was passed through a filter and concentrated under reduced pressure at <60 °C until a volume 400 mL of extract was obtained. This concentrated solution was dried under reduced pressure at < 70 °C, and then pulverized to obtain 98.2 g of the dried product, RW0117 (yield: 9.82%).

### 2.3. HPLC-UV/DAD Conditions

Quantitative analysis of RW0117 was performed using an Agilent 1260-DAD system (Santa Clara, CA, USA) and Inno column C18 (250 mm × 4.6 mm, 5 μm) Santa Clara, CA, USA). The mobile phase consisted of water containing 0.05% trifluoroacetic acid (A) and acetonitrile containing 0.05% trifluoroacetic acid (B). The gradient elution was as follows: 0–30 min of 30% (B), 30–31 min of 60–100% (B), 31–36 min of 100% (B), 36–37 min of 100–30% (B), 37–42 min of 30% (B) The post-running time was 10 min after restoration of the initial condition. The mobile phase flow rate was 0.7 mL/min and the injection volume was 10 μL. A PDA eλ detector was set to an absorbance of 340 nm for eupatilin, jaceosidin and, hispidulin. The peaks for eupatilin, jaceosidin and, hispidulin in RW0117 were compared with their respective standard compounds.

### 2.4. Cell Culture

RAW264.7 murine macrophage cells were obtained from the Korean Cell Line Bank (Seoul, Republic of Korea) and cultured at 37 °C in DMEM supplemented with 10% heat-inactivated fetal bovine serum (FBS) and 1% penicillin/streptomycin in a humidified atmosphere with 5% CO_2_ and 95% air. The cells were subcultured every 2 days to maintain monolayer cells.

### 2.5. Measurement of RW0117 Cytotoxicity on RAW264.7 Cells 

The cytotoxic effect of RW0117 in RWA264.7 cells was examined using an EZ-Cytox enhanced cell viability assay kit. Cells were seeded onto 96-well plates and treated with various concentrations of RW0117 for 24 h at 37 °C in a humidified atmosphere of 5% CO_2_ and 95% air.

### 2.6. Determination of Nitric Oxide, IL-6, and TNF-α Production

RAW264.7 cells were treated with RW0117 (200 μg/mL, 100 μg/mL, 50 μg/mL, 25 μg/mL, 12.5 μg/mL, 6.3 μg/mL, 3.1 μg/mL, 1.6 μg/mL, 0.8 μg/mL) for 2 h followed by treatment with LPS for 20 h in a CO_2_ incubator. The positive control consisted of macrophage cells treated with LPS alone for NO and IL-6 production. The supernatants were collected after centrifugation (900 rpm for 3 min) and frozen at −80 °C for further analysis. NO concentration in the supernatants was measured using a Griess reagent system (Promega, WI, USA), whereas IL-6 was measured using a kit for sandwich ELISA from eBioscience (San Diego, CA, USA), according to the manufacturer’s instructions.

### 2.7. Immunoblotting

RAW 264.7 cells were treated RW0117 (100 μg/mL, 50 μg/mL) for 2 h and then treated with LPS for 30 min (for phosphorylation of c-Jun, EKR, JNK, p38, p65) or 20 h (for protein expression of iNOS and COX-2). Following treatment, the cells were washed with cold PBS and lysed with cold radioimmunoprecipitation assay (RIPA) buffer containing a protease inhibitor cocktail (Roche Diagnostics Corp., Indianapolis, IN, USA), 1 mM dithiothreitol (Wako, Tokyo, Japan), 1 mM phenylmethylsulfonyl fluoride (Sigma), 1 mM sodium orthovanadate (Sigma), and 10 mM β-glycerophosphate (Sigma). Cell lysis, collection of supernatants, protein quantification, protein electrophoresis, protein transfer, and membrane development were all performed as described in our previous report [25,26]. The protein bands were visualized using an enhanced chemiluminescence system (GE Healthcare Life Sciences).

### 2.8. Real-Time Reverse Transcription Polymerase Chain Reaction 

RAW 264.7 cells were treated with RW0117 (100 μM, 50 μM) for 2 h, and then treated with LPS for 4 h. Following treatment, the cells were washed with PBS and lysed using the RNeasy mini kit (Qiagen, Valencia, CA, USA), after which, a procedure for total RNA purification was performed according to the manufacturer’s protocol. RNA was converted into cDNA using the RevertAid First Strand cDNA Synthesis kit (Thermo Scientific, Madison, WI, USA). To amplify the cDNA encoding for mouse IL-6, specific primers such as TNF-α and β-actin genes were used (Table 1). PCR amplification was performed using Power SYBR Green PCR Master Mix (Applied Biosystems, Foster City, CA, USA). Relative expression levels were determined using real-time reverse transcription PCR (qRT-PCR) by the Quant3 real-time PCR System (Applied Biosystems). Data were normalized to the amount of β-tubulin.

### 2.9. Immunofluorescence Microscopy

To investigate the localization of p65 in RAW264.7 cells treated with LPS or RW0117, the cells were seeded onto gelatin-coated coverslips (Paul Marienfeld GmbH & Co. KG, Lauda-Königshofen, Germany) in a 24-well plate, and grown to 70% confluence. Cells in the experimental group were treated with RW0117 (25 µg/mL and 50 µg/mL) for 2 h and then treated with LPS for 60 min. Subsequently, the cells were washed with PBS and fixed with 4 % formalin (pH 7.2) for 15 min. Additionally, cells were washed three times with PBS and incubated with blocking solution (5% BSA containing 0.1% Trion X-100) for 1 h. fter blocking, the cells were incubated with p65 antibody for 12 h at 4 °C, washed three times with PBS, and later incubated with Alexa Fluor 488-conjugated goat antirabbit antibody for 1 h in the dark. After incubation, the cells were again washed three times with PBS. The coverslips were removed from the 24-well plate and mounted onto glass slides containing a drop of mounting medium (DAPI, Vectashield, Burlingame, CA, USA). The fluorescent-labeled cells were analyzed using a confocal laser scanning microscope (Carl Zeiss Microscopy, Oberkochen, Germanay).

### 2.10. Animals

Male Sprague-Dawley rats (7 weeks old) (supplied by OrientBio, Seongnam, Korea) were used for all the experiments. The animals were allowed an acclimation period for 7 days under a controlled temperature of 22 °C ± 2 °C with a 12 h light/dark cycle and free access to food and water All animal experiments were performed in accordance with the instruction of the Ethics Comminttee for Use of Experimental Animals at Gachon University (Ethical approval code: 2019-032).

### 2.11. Ethanol and HCl-Induced Gastric Ulcer

Acute gastric lesions were induced by oral administration of 0.1 mL/20 g of a mixture containing 0.15 M HCl in 98% ethanol, a dose that induces significant gastric ulceration [27]. After 24 h of food deprivation, groups of animals (n = 5/group) were orally administered different doses of RW0117 (25 mg/kg and 50 mg/kg in 1.5% carboxymethylcellulose solution). Subsequently, 1 h after oral administration of the above solutions, the animals were orally administered 0.2 mL ethanol or the ethanol/HCl mixture, and 1 h later, they were euthanized. Their stomachs were removed, and then incised along the greater curvature. The stomachs were gently rinsed with saline solution to remove the gastric contents and blood clots.

### 2.12. Histological Analysis

A small portion of the stomach of each rat was fixed in 4% formalin solution for 24 h. The sections of the stomach tissue were dehydrated with graded concentrations of ethanol, passed through xylene, and embedded in paraffin. The paraffin sections were stained with periodic acid-Schiff stain [17].

### 2.13. Statistical Analysis

Results of the three independent experiments were expressed as mean ± standard deviation (SD). All statistical analyses were performed using one-way analysis of variance (ANOVA) followed by Tukey’s post hoc test. ** *p* < 0.01 or * *p* < 0.05 indicated significance. 

## 3. Results and Discussion

### 3.1. HPLC Profile of RW0117

**Figure 1:**
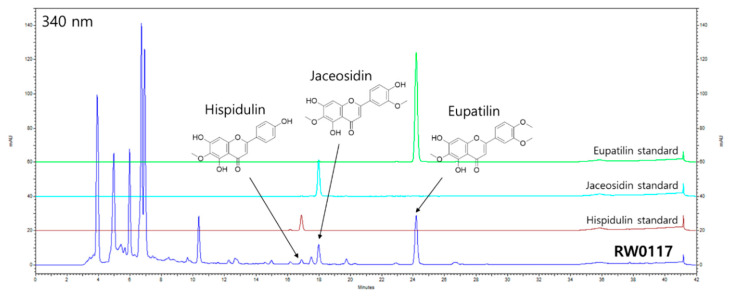
HPLC profiles of RW0117. The peak of eupatilin, jaceosidin, hispidulin at 340 nm in RW0117 were compared with those of their respective standard compounds.

**Figure 2:**
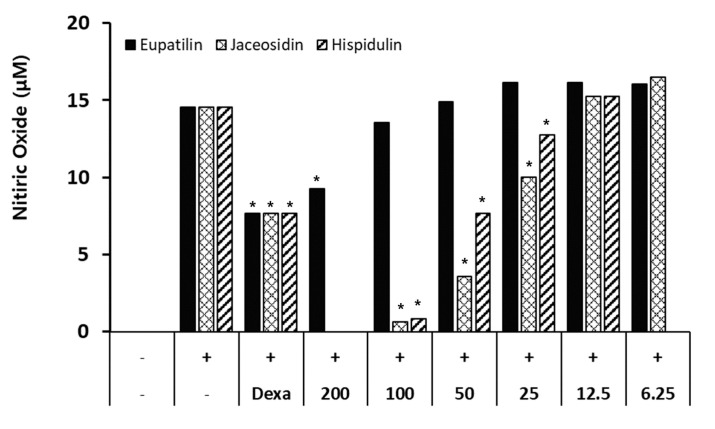
The inhibitory effects of eupatilin, jaceosidin and hispidulin on nitric oxide production in LPS-stimulated RAW264.7 cells; (−): vechicle group (0.1% DMSO-treated group), (+): positive control group (500 ng/mL of LPS treatment for 20 h), Dexa: dexamethasone for 2 h and then incubated with LPS for 20 h. Cells were treated with eupatilin or jaceosidin or hispidulin for 2 h and then incubated with LPS for 20 h. Results are expressed as mean ± SD of triplicate experiments. Statistical significance was determined using one-way analysis of variance (ANOVA) followed by Tukey’s post hoc test. * *p* < 0.001 vs. LPS treated group.

Through HPLC analysis we showed that RW0117 contained phytochemicals such as eupatilin, jaceosidin, and hispidulin (Figure 1). From HPLC chromatogram we calculated that RW0117 contained 0.573%, 0.223%, and 0.070% of eupatilin, jaceosidin and hispidulin, respectively (Table 2). Previous studies reported that these three compound showed anti-inflammatory activity in vitro and in vivo [22,23,24]. Therefore, we also confirmed eupatilin, jaceosidin, and hispidulin showed the inhibitory effects of nitric oxide production by LPS in RAW264.7 cells. As shown in Figure 2, treatement of eupatilin (200 μM), jaceosidin (200 μM, 100 μM, 50 μM, and 25 μM) and hispidulin (100 μM, 50 μM, and 25 μM) suppression of LPS-induced nitric oxide production in concentration dependent manner, respectively (Figure 2). In addition, these concentrations did not show cell cytotoxicity in RAW264.7 cells (data not shown). Taken together, it was predicted that RW0117 could exhibit anti-inflammatory activity because it contained eupatilin, jaceosidin, and hispidulin.

### 3.2. The Effects of RW0117 on Nitric Oxide Production in LPS-Stimulated RAW 264.7 Cells

**Figure 3:**
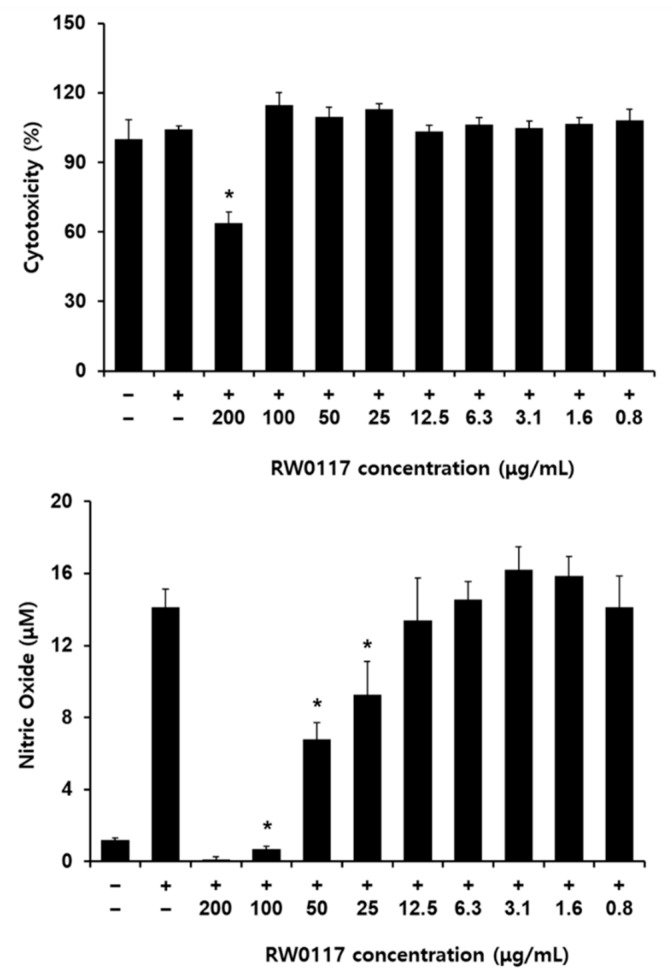
The effects of RW0117 on nitric oxide production in LPS-stimulated RAW264.7 cells; (−): vechicle group (0.1% DMSO-treated group), (+): positive control group (500 ng/mL of LPS treatment for 20 h). Cells were treated with RW0117 for 2 h and then incubated with LPS for 20 h. Results are expressed as mean ± SD of triplicate experiments. Statistical significance was determined using one-way analysis of variance (ANOVA) followed by Tukey’s post hoc test. * *p* < 0.001 vs. LPS treated group.

Treatment of RAW264.7 cells with LPS (an inflammatory substance) leads to the production of proinflammatory mediators (nitric oxide, IL-6, IL-1β, and TNF-α). To confirm the inhibition of LPS-induced inflammation by RW0117, we investigated the production of inflammatory mediators. First, the cytotoxicity of RW0117 was confirmed in the presence of LPS and RW0117. As described in the experimental method section, the cells were pretreated with RW0117 for 2 h and then incubated with LPS for 20 h. The cytotoxicity was confirmed in the group treated with 200 μg/mL of RW0117; however, no cytotoxicity was observed at a concentration of <200 μg/mL (Figure 3). Next, the inhibitory effect of RW0117 on nitric oxide (NO) production was analyzed. LPS-induced NO production was significantly inhibited at 25, 50, and 100 μg/mL of RW0117 treatment (Figure 3).

### 3.3. Analysis of the Effect of RW0117 on Cytokine Production and mRNA Expression in RAW264.7 Cells

**Figure 4:**
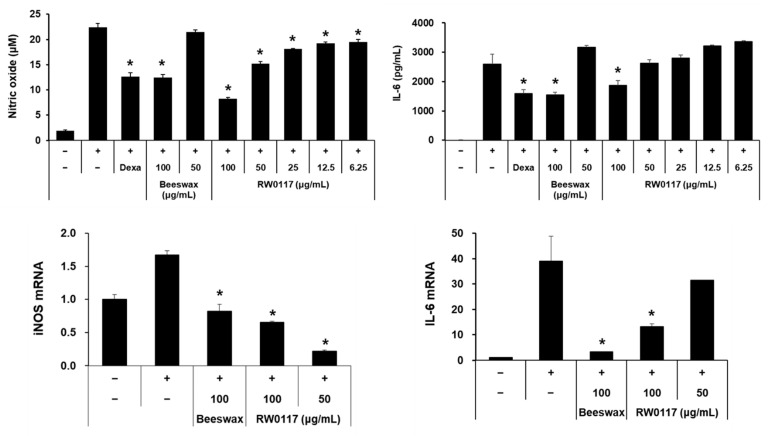
Effect of RW0117 on NO/IL-6 production and NO/IL-6 mRNA expression in LPS-stimulated RAW264.7 cells; (−): nontreated group, (+): group treated with 500 ng/mL of LPS, Dexa: dexamethasone-treated group, beeswax: beeswax alcohol-treated group. Cells were treated with either dexamethasone, beeswax, or RW0117 for 2 h and then incubated with LPS for 20 h. Results are expressed as mean ± SD of triplicate experiments. Statistical significance was determined using one-way analysis of variance (ANOVA) followed by Tukey’s post hoc test. * *p* < 0.005 vs. LPS treated group.

The inhibitory effects RW0117 against NO and IL-6 production were analyzed using NSAID (dexamethasone, Dexa.) and beeswax alcohols (Beeswax) (Abexol^®^, Rainbow & Nature Pty Ltd., Seoul, Korea). as positive controls. Beeswax (a mixture of six high molecular weight aliphatic alcohols purified from beeswax), has been shown gastroprotective effects in experimental and clinical studies [28]. As shown in Figure 4, NO and IL-6 production induced by LPS was significantly inhibited in the group treated with Dexa. as the positive control. Additionally, in the group treated with 100 μg/mL of beeswax alcohol, the inhibitory activity due on NO and IL-6 production displayed similar with the Dexa. group. In the group treated with 50 μg/mL of beeswax alcohol, no inhibitory activity was observed. Conversely, in the case of RW0117, there was a significant inhibitory effect on LPS-induced NO production at all concentrations (6.3–100 μg/mL), while IL-6 production was significantly inhibited at 100 μg/mL of RW0117 (Figure 4). Next, the mRNA expression of iNOS and IL-6, which are involved in NO and IL-6 production, were analyzed. The effect of LPS on mRNA expression was significantly suppressed following treatment with RW0117 (Figure 4). These data suggested that RW0117 possessed anti-inflammatory activity related to NO and IL-6 production in RAW264.7 cells. In addition, treatment with RW0117 in low concentration (50 μg/mL) showed more effective than beeswax alcohol.

### 3.4. Analysis of the Effect of RW0117 on MAPK and NF-kB Signaling Pathways

**Figure 5:**
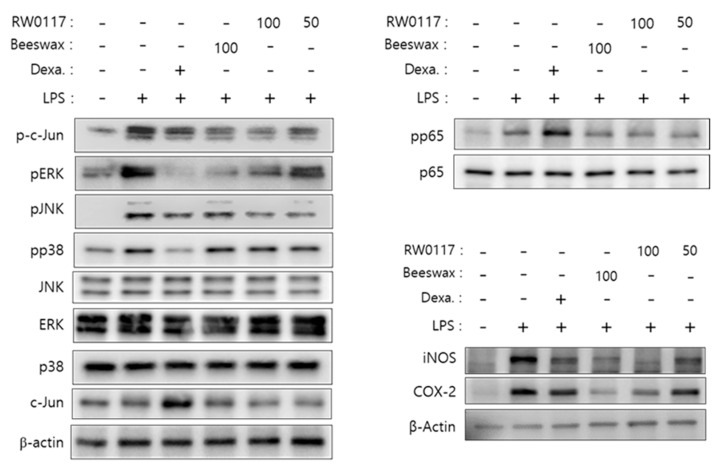
Effect of RW0117 on the phosphorylation of several signaling proteins in LPS-stimulated RAW264.7 cells; (−): DMSO (0.05 %)-treated group, Beeswax: beeswax alcohol-treated group, Dexa.: dexamethasone-treated group, (+): group treated with 500 ng/mL of LPS. Whole cell-lysates were immunoblotted with the indicated specific-antibodies. β-Actin served as an internal loading control.

To analyze the intracellular signal pathways involved in anti-inflammatory activity of RW0117 in RAW264.7 cells, we examined LPS related signaling pathways proteins. First, phosphorylation of MAPK, a signaling protein representative of ERK, JNK, and p38, activated by LPS, was confirmed by western blotting. As shown in Figure 5, LPS treatment strongly induced ERK, JNK, and p38 phosphorylation in RAW264.7 cells. However, ERK and JNK phosphorylation was inhibited by treatment of RW0117 at a concentration of 100 μg/mL and 50 μg/mL. A strong phosphorylation inhibitory activity, similar to that of the positive controls (Dexa. or beeswax alcohol) was observed in the group treated with 100 μg/mL of RW0117. However, it did not affect the phosphorylation of p38. In addition, phosphorylation of the transcription factor c-Jun, which is activated by the phosphorylation of MAPK, was examined. The phosphorylation of c-Jun was strongly induced by treatment with LPS, and it was confirmed that this phosphorylation was inhibited by treatment with 100 μg/mL of RW0117 (Figure 5).

Next, the NF-κB pathway, an inflammatory pathway activated by LPS, was analyzed. When cells are in normal state, IκBα protein exists in the cytoplasm as a complex with p65 and p50. However, when stimulated by LPS, IκBα undergoes phosphorylation followed by decomposition. Simultaneously, phosphorylation of p65 occurs and the phosphorylated protein is transported to the nucleus, where it acts as a transcription factor. As shown in Figure 5, phosphorylation of p65 was confirmed when the cells were treated with LPS. In addition, phosphorylation of p65 was slightly suppressed when treated with beeswax alcohol. LPS-induced phosphorylation was also slightly suppressed by treatment with RW0117. However, dexamethasone did not inhibit p65 RW0117. Moreover, phosphorylation of iNOS and COX-2 proteins was strongly inhibited by RW0117 (Figure 5). Based on these results, it was predicted that RW0117 inhibits the production of inflammatory factors such as NO, IL-6, and COX-2 by inhibiting the JNK/ERK pathway and activity of the transcription factor c-Jun rather than inhibiting phosphorylation of the NF-κB pathway (Figure 5).

### 3.5. Analysis of Nuclear Translocation of NF-kB p65 by RW0117 in RAW264.7 Cells

**Figure 6:**
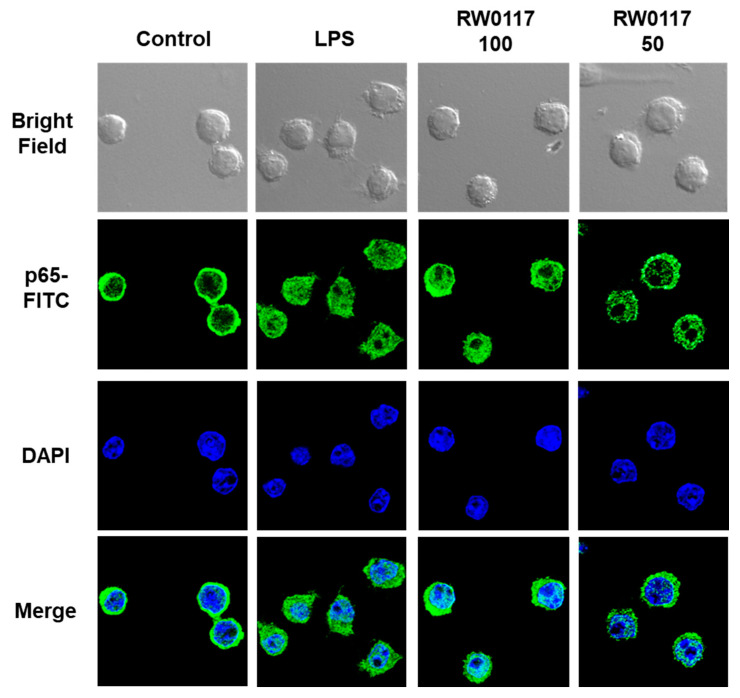
RW0117 inhibits LPS-induced p65 nuclear translocation in RAW264.7 cells. RAW264.7 cells were seeded onto 0.1% gelatin-coated coverslips for overnight. After that beeswax alcohol (100 μg/mL) or RW0117 (100 μg/mL, 50 μg/mL) treated for 2 h, then stimulated with LPS (1 μg/mL) for 1 h. RAW264.7 cells were fixed and immunostained with anti-p65-FITC antibody and the nucleus was counterstained with DAPI. The fluorescence images were acquired using ZEN 2.3 confocal imaging software. LPS was used as a positive control for activation of RAW264.7 cells.

When RAW264.7 cells were treated with LPS, phosphorylation of p65 and its nuclear translocation were observed. As shown in Figure 6, analysis via fluorescence staining demonstrated that nuclear translocation of p65 stained with fluorescein-5-isothiocyanate (FITC) occurs due to treatment with LPS. However, when cells were treated with beeswax alcohol and RW0117 for 2 h and then treated with LPS, it was confirmed that the nuclear translocation of p65 was suppressed compared to that observed in the group treated with LPS alone. These results show that RW0117 weakly inhibits the LPS-induced NF-κB signaling pathway, and beeswax alcohol shows stronger inhibition than RW0117. This result is consistent with the inhibition of p65 phosphorylation as shown in Figure 5.

### 3.6. Protective Effects of RW0117 in Rats with HCl/EtOH-Induced Gastric Ulcers

**Figure 7:**
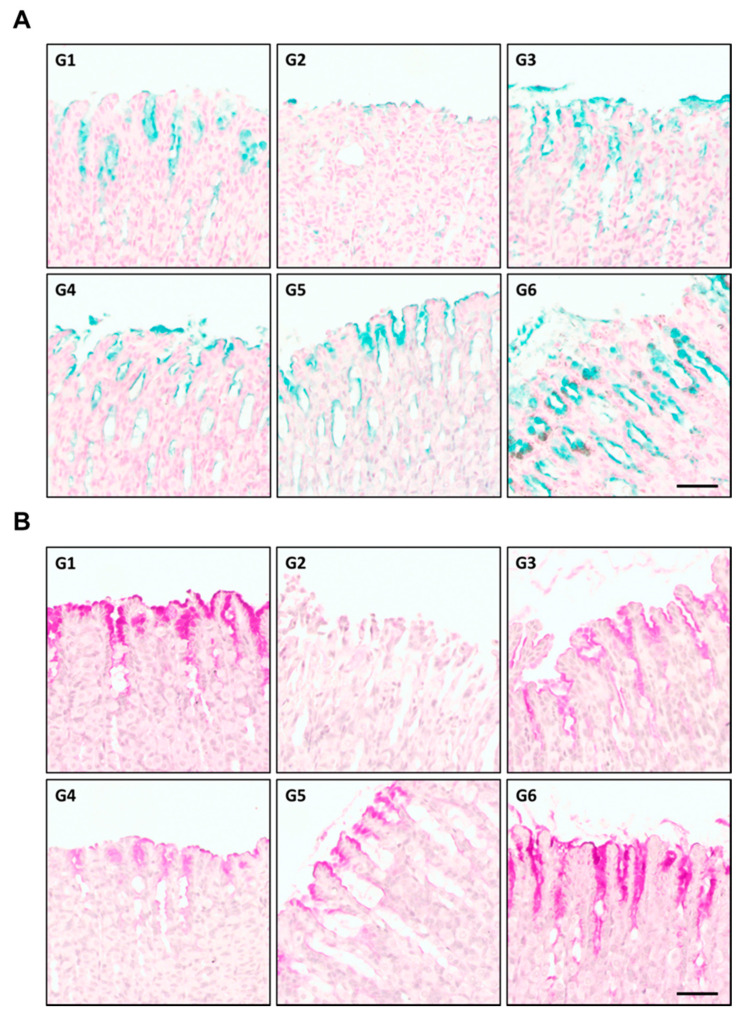
Alcian/periodic acid-Schiff staining of HCl-EtOH induced ulceration in rat stomach. Upper pictures (G1–G6): Alcian staining for acidic mucin, Lower pictures (G1-G6): Periodic acid-Schiff staining for acidic/neutral mucin; G1: Normal group, G2: HCl/EtOH treated group, G3: omeprazole, G4: beeswax alcohol 100 mg/kg G5: low-dose of RW0117 (25 mg/kg), G6: high-dose of RW0117 (50 mg/kg) (scale bar = 50 μm). (**A**) Alcian blue staining of HCl-EtOH induced ulceration in rat stomach. (**B**) Periodic acid-Schiff staining of HCl-EtOH induced ulceration in rat stomach.

Ethanol is a well-known damaging agent to the gastric mucosa, and excessive ethanol ingestion, serving as the main inducer of gastric ulcers in humans, causes acute gastric mucosal damage. After inducing stomach ulcers with HCl/EtOH, the rats were sacrificed and their stomachs were excised. The amount of mucin was analyzed using periodic acid-Schiff staining [17]. Compared to that in the normal group (G1), the amount of acidic mucin (blue) in the group with gastric ulceration (G2) was significantly reduced. However, it increased in the positive control group treated with omeprazole + HCl/EtOH (G3) and in the beeswax alcohol 100 + HCl/EtOH (G4) group, compared to that in the G2 group. Similar results were obtained in the RW0117-treated groups G5 (RW0117: 25 mg/kg) and G6 (RW0117: 50 mg/kg); the stomachs of rats treated with low-dose RW0117 (G5) showed similar amounts of acidic mucin compared to those in the positive control group, and the amount of mucin in the high-dose group (G6) was significantly increased (Figure 7A).

Similar to the results obtained with alcian blue staining, the amount of acidic/neutral mucin (magenta) significantly decreased in the group with gastric ulceration (G2) compared to the normal group (G1). The omeprazole-treated positive control group (G3) showed a significant increase in acidic/neutral mucin, and the beeswax alcohol-treated group (G4) showed a slight increase. The mucin level in the gastric mucosa of the low-dose RW0117 group (G5) was similar to that of the positive control group. The amount of mucin in the gastric mucosa of the high-dose RW0117 group (G6) showed a similar significant increase to that of the gastric mucosa of the G1 group (Figure 7B).

### 3.7. Analysis of the Gene Expressions of MUC1, MUC5AC, and MUC6 in Gastric Ulcer in Rats

**Figure 8:**
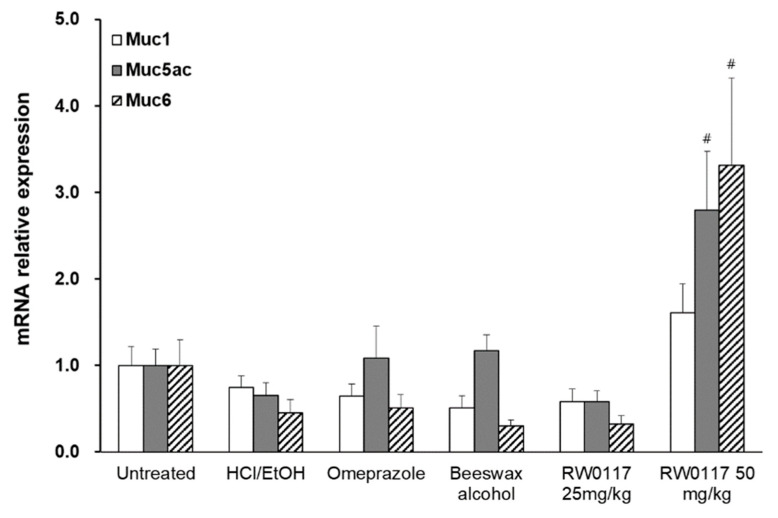
Analysis of mRNA expression of Muc1, Muc5ac, Muc6 in ulcer-induced rat stomach. Untreated: nontreated group, HCl/EtOH: HCl/EtOH-treated group, Omeprazole: omeprazole + HCl/EtOH-treated group, beeswax alcohol: beeswax alcohol 100 mg/kg + HCl/EtOH treated group, RW0117 25 mg/kg: RW0117 25 mg/kg + HCl/EtOH-treated group, RW0117 50 mg/kg: RW0117 50 mg/kg + HCl/EtOH-treated group. Statistical significance was determined using one-way analysis of variance (ANOVA) followed by Tukey’s post hoc test. # *p* < 0.05 vs. control group.

To verify the results obtained after staining, the expression of genes, such as MUC1, MUC5AC, and MUC6, in the stomach tissue was measured. The HCl-EtOH-induced gastric ulcer group (control) showed a decrease in the expression of MUC1, MUC5A, and MUC6 genes compared to the normal group (normal). In the control groups treated with omeprazole, beeswax alcohol 100 mg/kg, and low-dose of RW0117 (25 mg/kg), the decrease in gene expression was not reversed. However, the expression of MUC5AC in the positive control group was recovered to the same level as that of the normal group (omeprazole, beeswax alcohol). The group treated with high-dose RW0117 (50 mg/kg) showed an increase in the expression level of MUC1 compared to the control group, and the gene expression of MUC5AC and MUC6 increased significantly. These results, together with the histopathology results described above, show that a different pathway may be involved in the protective action of beeswax alcohol 100 and RW0117 on the gastric mucosa other than increasing the expression of genes encoding mucin. In addition, the group treated with high-dose RW0117 showed a significant increase in mucin, as per the histopathology and molecular biology findings; therefore, it was inferred that it is more directly involved in the protection of the gastric mucosa through the secretion of mucus from the gastric mucosa than that observed in the other groups. In addition, it is expected that further studies are needed on the fact that omeparzole, a drug that acts as a proton pump inhibitor, did not affect muc1 and muc6 mRNA expression. (Figure 8).

## 4. Conclusions

Medicinal herbs have recently attracted attention as health beneficial foods and source materials for drug development. Previous studies demonstrated that *A. argyi* have various physiological functions such as antioxidant, antitumor, anti-inflammatory, anticoagulant, antiosteoporotic activities, and immunomodulation. These functions correlated with flavones, organic acid, terpens, polysaccharides, and coumarins isolated from *A. argyi* [29,30,31,32,33]. 

In this study, we confirmed RW0117 showed anti-inflammatory activity such as inhibition of NO/IL-6 production and iNOS/COX-2 protein expression mediated by suppression of ERK-JNK/c-Jun signal pathways in RAW264.7 cells. In addition, in vivo results indicated that treatment with high doses of RW0117 (50 mg/kg) helped recover certain amounts of acidic and neutral mucin and the expression levels of genes encoding mucin in rat models of acute gastric ulcer using HCl/ethanol. These results indicated that RW0117 can protect HCl/ethanol-induced rat from gastric mucosal injury through inhibiting inflammatory response. Furthermore, it can be predicted that eupatilin, jaceosidin, and hispidulin in RW0117 will be involved in the activity.

Collectively, these results indicated that RW0117 (*A. argyi* extract) has strong anti-inflammatory and antigastric ulcer activity and can be developed as a therapeutic agent or dietary supplement for stomach ulcers in future.

## Figures and Tables

**Table 1:** The primer sets used for real-time PCR analysis.

Gene	Forward Primer (5′-3′)	Reverse Primer (5′-3′)
Mouse IL-6	GAGGATACCACTCCCAACAGACC	AAGTGCATCATCGTTGTTCATACA
Mouse iNOS	ACATCGACCCGTCCACAGTAT	CAGAGGGGTAGGCTTGTCTC
Mouse β-tubulin	CTCCCAGGTTAAAGTCCTTC	GCAACATAAATACAGAGGTG
Rat MUC1	Rn01462585_m1 (Muc1)	
Rat MUC5AC	Rn01451252_m1 (Muc5ac)	
Rat MUC6	Rn01759814_m1 (Muc6)	
Rat GAPDH	Rn01775763_g1 (gapdh)	

**Table 2:** Contents of representative compounds in RW0117.

Active Compound	Content (%)
Eupatilin	0.573 ± 0.002
Jaceosidin	0.223 ± 0.006
Hispidulin	0.070 ± 0.002

## Data Availability

Not applicable.

## Supplementary Materials

The following are available online at https://www.mdpi.com/2223-7747/10/2/332/s1, Table S1: DNA sequencing methods and results of *Artemisia argyi*.
