# Supplementary material for: Anti-Inflammatory Effect of Artemisia argyi on Ethanol-Induced Gastric Ulcer: Analytical, In Vitro and In Vivo Studies for the Identification of Action Mechanism and Active Compounds
Source: Plants (Basel). 2021 Feb 9;10(2):332. doi: 10.3390/plants10020332 (PMC7914715; doi:10.3390/plants10020332)
Supplement: Supplementary file 1 [file plants-10-00332-s001.pdf]

Supplementary table 1.

Material & Method

Genoimic DNA extraction from the leaves was performed using the G-spinTMllp Genominc DNA extraction Kit (Intron, Korea) then purified PCR products using PCRquick-spin™ PCR product Purification Kit (Intron, Korea). To identified plant species, chloroplast DNA marker (*matK, rbcL, atpF-atpH, psbK-psbI, trnH-psbA*) and nuclear DNA marker ITS (intergerc transcribed spacer) were amplified. The final nucleotide sequence was analyzed using Geneious 11.1.2.

Result

The result of comparing the base sequence with Genbank using BLAST is as follows Table 1. As a result of BLAST analysis of the nrlTS nucleotide sequence of the sample, it was found to be 99% consistent with *Artemisia argyi L.*, thus it was confirmed.

Table 1. GenBanK BLAST Report

**rbcL**	**matK**	**ITS**
Artemisia argyi<br>1299[i] 100%[ii] KM386991.1[iii]	Artemisia argyi<br>1572[i] 99%[ii] KM386991.1[iii]	Artemisia argyi<br>1269[i] 99%[ii] KX421699.1[iii]
Artemisia montana<br>1299[i] 100%[ii] KF887960.1[iii]	Artemisia montana<br>1572[i] 99%[ii] KF887960.1[iii]	Artemisia argyi<br>1269[i] 99%[ii] KX421696.1[iii]
Artemisia argyi<br>1299[i] 100%[ii] GQ436428.1[iii]	Artemisia argyi<br>1567[i] 100%[ii] KR231888.1[iii]	Artemisia argyi<br>1269[i] 99%[ii] GU724269.1[iii]
**atpF-atpH**	**psbK-psbI**	**trnH-psbA**
Artemisia annua<br>1003[i] 100%[ii] MF623173.1[iii]	Artemisia argyi<br>758[i] 99% [ii] KM386991.1[iii]	Artemisia argyi var. gracilis<br>859[i] 100%[ii] KU555808.1iii[iii]
Artemisia gmelinii<br>1003[i] 100%[ii] KY073390.1[iii]	Artemisia montana<br>758[i] 99% [ii] KF887960.1[iii]	Artemisia argyi var. gracilis<br>859[i] 100%[ii] KU555808.1[iii]
Artemisia argyi<br>1003[i] 100%[ii] KY085890.1[iii]	Artemisia montana<br>752[i] 99% [ii] NC_037388.1[iii]	Artemisia argyi<br>859[i] 100%[ii] KU555799.1[iii]

[i)]Maximum match Scores [ii)]Maximum similarities [iii)]Accession Num.

Reference

Liu, Geyu, et al. "Evaluation of DNA barcode candidates for the discrimination of Artemisia L." Mitochondrial DNA Part A 28.6 (2017): 956-964.

Wang, Xiao-yue, et al. "ITS2, a Better DNA Barcode than ITS in Identification of Species in Artemisia L." Chinese Herbal Medicines 8.4 (2016): 352-358
